# A System of Phrenology

**Published:** 1831-04-01

**Authors:** 


### COMBE'S PHRENOLOGY.

A SYSTEM OF PHRENOLOGY. By *George Combe*. Third Edition. Anderson, Edinburgh; Longman, London; pp. 724, seventy-one Figures. MDCCCXXX.

"Res non verba quæro."

OBSERVING the great increase of phrenologists yearly, and the extraordinary diffusion of their doctrines, we naturally conclude that the charge must be unjust, which represents phrenology as a deceptive system, contaminated with an excess of folly, mischief, and irreligion: analogy and experience, history and philosophy, abound with evidences of the improbability—that *very many* intelligent, upright, and learned persons should become so infatuated as to approve the tenets of such a system: entertaining this sentiment, therefore, we have more than once* endeavoured to procure, for the phrenological doctrines, a fair hearing and judgment.

Dr. Gall discovered phrenology: Dr. Spurzheim improved it vastly: Mr. Combe has matured it; and in his "*System*" its principles maintain the precision and dignity of pure inductive science: every page of his elaborate and instructive work, indeed, exhibits the admirable features of a mind evidently powerful, discriminative, and just.

We have said heretofore, and we now repeat deliberately, our entire persuasion, that phrenology includes not a few subjects most worthy of contemplation and research for the medical philosopher: it reveals very beautifully many of the mysterious re-actions of body and mind, in health and disease. Beyond all others, the medical observer enjoys frequent and valuable opportunities of ascertaining the exactness of the phrenological proposition—that the brain constitutes a system of distinguishable organs, every ONE of which, as a material instrument, co-exists, and co-operates, with a determinate ONE of the mind's aggregated faculties; and that, consequently, from structural or functional lesion, of *one, some, or several* of the cerebral organs, the MANIFESTATIONS of *one, some, or several* of the correlative mental faculties, may be deranged, and thus originate modifications of insanity. Now, were it for no higher object than that of gaining a mere speculative accomplishment, we should essay to cultivate a knowledge of the mind's faculties and the organ proper to each of them in the brain, according to their specific distinctions in phrenology, *(for, like other sciences, this cannot be tried fairly except by its own laws,)* so as, with this knowledge, we may be enabled to mark the altered *manifestations* of faculties in disease and insanity, and to ascertain the condition of corresponding organs as this shall be revealed by necrotomical investigation. Numerous and diversified observations, in this way, will prove or disprove the phrenological doctrine, promote a true mental philosophy, and intro-

* Med. Chirurg. Review, March, 1823; March, 1824; and October, 1826.

duce greater precision into the details of cerebral anatomy, physiology, and pathology.

Mr. Combe, in all his writings, discusses the questions having relation to the phrenological science, in a manner unusually explicit and generous: he distinguishes its principles also with illustrations and evidences unusually appropriate and conclusive. On this occasion, we are not required to examine anew his doctrines, and their inherent tendency to facilitate education, metaphysics, morals, and legislation: nevertheless, we may particularize—as subjects adapted to enlighten medical students—his sections on *plurality of the mental faculties and their organs—size of organs, their health, exercise, temperament, and disease—functions of the nerves and spinal marrow—the brain, cerebellum, and skull—integuments of the brain—bones of the skull—frontal sinus—length, breadth, and forms of the cerebral organs—brains of the lower animals—national character and development of brain—materialism—and injuries of the brain*—all which subjects are elucidated with an impressive and perspicuous eloquence, every-way dissimilar from the vapouring of those sciolists who, with the blaze of fancy, obscure the brilliancy of TRUTH.

Such, then, being the character of Mr. Combe's philosophy, and such the topics in his "*System*" for which we solicit the regard of our profession, we would say emphatically to the medical inquirer desirous of investigating the relations of man's organic and mental natures—bring with you a mind clear from all preconceptions and hypothetical notions; extricated from the debasing trammels of authority; unblighted by the poison of prejudice: never impute error to doctrines you have not taken due means to understand; never seek to repel the validity of inductions you yourself do not know to be defective, and never question the accuracy of experiments you have not repeated and found to be inexact: while occupied with the examination of notional or demonstrable questions, weigh your thoughts well; scan all arguments with composure and manliness; consider deeply in what degree your ultimate sentiments may influence truth; and never, never contend exclusively in a discussion for the victory; whoever has, at any time, suffered himself to be so bewildered by the tumult of headlong or ambitious emotion, as to enjoy the vain-glory of triumphing in a speculative wrangle, has as certainly been doomed, in after-days, to endure the bitterness of repentance for having been the means of darkening counsel by words without knowledge: finally, in the exercise of reflection and judgment, maintain that mental and moral liberty which the CREATOR destined to be the high distinction of our race; with himself rests the cause when, in thought and action, man is most free—free as the billows of the great ocean, unfettered as the lightning of the sky.

